# First electromyographic monitoring of a progressive phrenic nerve palsy in a pulsed field ablation procedure

**DOI:** 10.1016/j.hrcr.2024.04.004

**Published:** 2024-04-10

**Authors:** Frederic Franceschi, Linda Koutbi, Baptiste Maille, Jean-Claude Deharo

**Affiliations:** ∗ Department of Cardiology, CHU Timone, Marseille, France; † Aix-Marseille University, Marseille, France; ‡ Center for CardioVascular and Nutrition Research (C2VN), INSERM, INRA, Marseille, France

**Keywords:** Pulsed field ablation, Phrenic nerve palsy, Compound motor action potential, Farapulse, Atrial fibrillation, Ablation, Complication

**Key Teaching Points**

• Sequential diaphragmatic compound motor action potential monitoring is feasible in pulsed field ablation (PFA) procedures.
• The onset phrenic nerve palsy pattern we observed was progressive, with a dose-effect relationship.
• When the phrenic nerve was paralyzed, vigorous diaphragmatic contractions were still observed during right-sided PFA applications, suggesting a phrenic nerve activation under the level of the conduction block by PFA.

## Introduction

Right phrenic nerve palsy (PNP) is a common complication of atrial fibrillation ablation procedures, in particular with cryoballoon.^1^ PNP is a specific complication of pulsed field ablation (PFA) either.^2^^,^^3^ Several cases of persistent PNP have been described.^2^^,^^3^ It should be noted that no phrenic nerve monitoring was performed in these studies; thus PNP incidence could have been underestimated. Moreover, nothing is known about the mechanism and the mode of occurrence of this complication. What is the onset pattern (sudden or progressive)? Is it dose dependent? Can-it be prevented? Several years ago, we developed a specific right phrenic nerve monitoring approach with compound motor action potential (CMAP) during cryoballoon procedures.^4^ We decided to perform systematic diaphragmatic CMAP monitoring in PFA procedures.

## Case report

A 75-year-old man was referred to our institution for atrial fibrillation (AF) ablation. He had multiple episodes of persistent AF complicated by heart failure and was maintained in sinus rhythm with amiodarone for several months. Moreover, he had a history of hypertension. Weight and height were 70 kg and 174 cm, respectively. The echocardiogram showed no left ventricular dysfunction in sinus rhythm. The left atrium was not enlarged (30 mL/m^2^).

**Figure 1:**
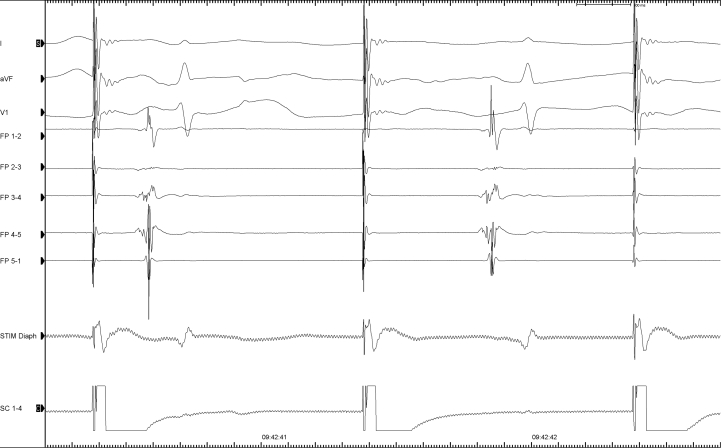
Right superior pulmonary vein (RSPV) and diaphragmatic compound motor action potential (CMAP) recording at baseline. Right phrenic nerve monitoring sequence with CMAP recording at baseline, before RSPV applications. These are tracings from the electrophysiology workstation, sweep speed 100 mm/s. The 3 upper lines are electrocardiogram leads I, VF, and V_1_. The 5 lines below are PV recordings with 31 mm FARAWAVE (Boston Scientific, St. Paul, MN) in the basket shape. The last line is the diaphragmatic CMAP recording. QRS far-field is visible in between the CMAPs.

**Figure 2:**
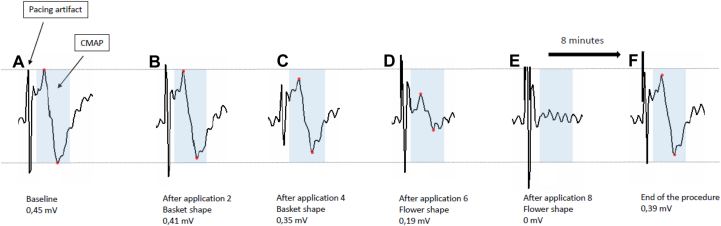
Diaphragmatic compound motor action potential (CMAP) evolution during right superior pulmonary vein (RSPV) ablation. Recordings from the electrophysiology workstation. Sweep speed is 100 mm/s. The first part of each recorded sequence is the pacing artifact. Its amplitude is variable, depending on respiratory movements. Diaphragmatic CMAP is seen after the pacing artifact. This is a biphasic signal with a positive-negative pattern. A red dot was placed at the positive and the negative peak; the CMAP area was shaded blue for easier reading. **A:** Baseline CMAP. Amplitude is 0.45 mV. **B:** CMAP after 2 applications in basket shape. Amplitude is 0.41 mV. **C:** CMAP after 4 applications in basket shape. Amplitude is 0.35 mV. **D:** CMAP after 6 applications (4 basket and 2 flower shape). Amplitude is 0.19 mV. In A, B, C, and D, diaphragmatic contraction was present during abdominal palpation. **E:** CMAP after 8 applications (4 basket and 4 flower shape). CMAP is no longer visible. Diaphragmatic contraction is absent during abdominal palpation. **F:** CMAP at the end of the procedure (8 minutes after the palsy occurrence). Incomplete recovery with CMAP amplitude at 0.39 mV. Diaphragmatic contraction is present during abdominal palpation.

**Figure 3:**
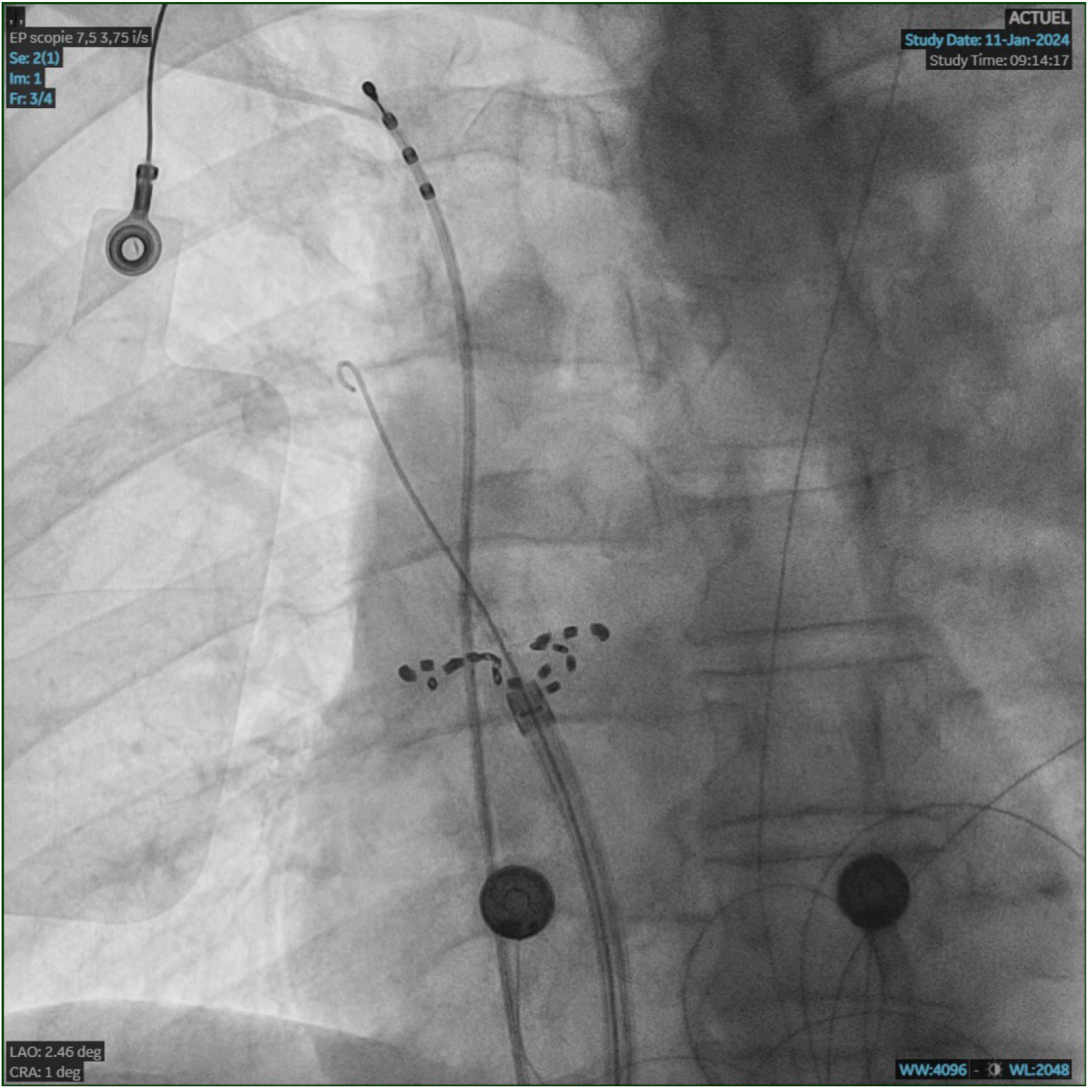
Catheter positioning during right superior pulmonary vein (RSPV) application. Anteroposterior fluoroscopic view. The J wire is inside the RSPV. The ablation catheter (FARADRIVE 31mm; Boston Scientific, St. Paul, MN) is in flower shape at the RSPV antrum. Note that no petal is inside the vein. The pacing catheter is at the junction of the superior vena cava – right subclavian vein.

We decided to perform AF ablation with PFA, using the FARAPULSE^TM^ system (Boston Scientific, St. Paul, MN). The procedure was done under general anesthesia with orotracheal intubation. The anesthesia protocol consisted of propofol and remifentanil infusion. Importantly for right phrenic nerve monitoring, curare was not used. A triple right femoral vein puncture was performed. A quadripolar Josephson Curve catheter (Response^TM^; Abbott, Chicago, Illinois) was positioned on the His bundle, and a steerable quadripolar deflectable catheter (Xtrem^TM^; MicroPort, Shanghai, China) was placed in the coronary sinus by femoral access. After transseptal puncture, the FARADRIVE^TM^ sheath (Boston Scientific) was advanced in the left atrium. At first, left pulmonary vein isolation was performed, and then the 31 mm FARAWAVE^TM^ was advanced to the right superior pulmonary vein (RSPV). The quadripolar Josephson Curve catheter was then positioned into a subdiaphragmatic hepatic vein, as previously described.^5^^,^^6^ Bipolar electromyographic signals were recorded between the proximal and distal electrodes. Signals were amplified and bandpass filtered between 5 and 150 Hz. After left-sided vein treatment, the steerable quadripolar catheter was relocated from the coronary sinus to the superior vena cava (SVC), to pace the right phrenic nerve at 60 pulses per minute (bipolar stimulation between proximal and distal electrodes with maximal output of 12 V at 2.9 ms). CMAP amplitude was measured peak to peak on the electrophysiology workstation. Baseline CMAP amplitude was obtained before the first right-sided application. Then CMAP amplitude was measured before each new FARAWAVE positioning. As shown in Figures 1 and 2, baseline CMAP amplitude was 0.45 mV. After 2 PFA applications in a basket shape, CMAP amplitude decreased to 0.41 mV (-9% from baseline). Two more applications were performed in basket shape after 30° rotation. CMAP amplitude decreased to 0.35 mV (-22% from baseline). At this time no alteration of diaphragmatic contraction was perceived with abdominal palpation. Then the FARAWAVE was set in the flower shape (Figure 3) and 2 more applications were done at the RSPV. CMAP decreased to 0.19 mV (-58% from baseline). Diaphragmatic contraction remained present at abdominal palpation. A 30° rotation of the catheter was made, and another 2 applications were performed. At this time CMAP disappeared completely, and diaphragmatic palsy was confirmed with abdominal palpation and fluoroscopy. In the hypothesis of a pacing catheter dislodgement, the Xtrem position was checked at fluoroscopy. We found no variation in catheter placement from the beginning of right-sided applications. Nevertheless, the pacing catheter was moved in the SVC with the aim to capture the right phrenic nerve, but it was no longer possible, thus confirming the occurrence of a paralysis. Moreover, at fluoroscopy we could no longer observe spontaneous diaphragmatic movements with breathing either. Interestingly, during all PFA applications at the RSPV, we could observe a vigorous diaphragmatic contraction. Moreover, during the PNP, the FARAWAVE was moved to the right inferior pulmonary vein, where several PFA applications were performed (4 in basket shape and 8 in flower shape). Vigorous diaphragmatic contractions were observed during the PFA applications, even though it was impossible to stimulate the phrenic nerve with the quadripolar catheter located in the SVC. Finally, the posterior wall and the roof were treated (16 PFA applications). At the end of the procedure (8 minutes after complete palsy occurred), we observed an incomplete recovery of the CMAP at 0.39 mV (-11% from baseline).

The next day, chest radiography showed no elevation of the right diaphragmatic dome and the patient described no dyspnea.

## Discussion

We report a case of transient phrenic nerve palsy during a PFA procedure where the right phrenic nerve was sequentially monitored with CMAP. We observed a progressive onset of the palsy with a dose-effect relationship. The more PFA was delivered at RSPV, the more phrenic nerve conduction was altered and CMAP amplitude decreased.

PFA applications are only a few milliseconds, but numerous applications have to be performed at each pulmonary vein. Each shot represents 5 PFA applications, and a minimum of 8 shots are currently recommended by the manufacturer. Thus each vein receives a minimum of 40 PFA applications. As a consequence, diaphragmatic CMAP monitoring is feasible in between shots, sequentially.

Our case is a transient PNP, but cases of persistent or permanent PNP have been reported.^7^

Here CMAP was recorded with a hepatic catheter. Most teams perform CMAP monitoring noninvasively with surface electrodes.^1^ Nevertheless, even if hepatic recordings are easier to read in real time,^5^ CMAP amplitude evolution is the same when comparing surface vs hepatic recordings simultaneously.^5^ Thus the progressive phrenic nerve palsy seen here would have been the same with surface electrode CMAP recording.

In our case, we decided to continue the PFA applications despite a decrease in CMAP amplitude and the occurrence of a palsy. This attitude is questionable. If we can later confirm that a decrease in CMAP amplitude is predictive of a palsy with further PFA applications, it will probably be recommended to stop the PFA applications if CMAP amplitude decreases. However, this limitation in PFA applications could have a negative impact on the durability of pulmonary vein isolation and thus the procedural long-term success.

Phrenic nerve pacing in the SVC with deflectable catheters is known to be unstable.^5^ In the case of CMAP monitoring during PFA procedures, the risk of pacing catheter instability and dislodgement is major owing to the large shakes generated by PFA applications. This underlines the need for a very stable phrenic nerve stimulation catheter, in order to achieve reliable monitoring. A dedicated catheter designed to pace the right phrenic nerve with a stable shape would be helpful.

Importantly, at the time of the phrenic nerve palsy, a vigorous diaphragmatic contraction was still present during right superior and right inferior pulmonary vein PFA applications. It should be explained by the fact that the right phrenic nerve is probably activated by PFA under the level of the conduction block. This fact can be falsely reassuring for operators.

## Conclusion

We report a case of transient right PNP during a PFA procedure. Sequential phrenic nerve monitoring with CMAP made it possible to highlight a progressive onset of paralysis. If confirmed in a larger cohort, this would call for systematic phrenic nerve CMAP monitoring in PFA procedures, which could help prevent the occurrence of diaphragmatic paralysis.

## Disclosures

Frédéric Franceschi is consultant for the company Circle-Safe. Linda Koutbi, Baptiste Maille, and Jean-Claude Deharo have no conflict of interest.
